# Ribosome profiling shows variable sensitivity to detect open reading frames for conventional and different types of cryptic T cell antigens

**DOI:** 10.1016/j.omtm.2024.101391

**Published:** 2024-12-06

**Authors:** Kyra J. Fuchs, Sofia Thomaidou, Arno R. van der Slik, Marian van de Meent, Peter A.C. ‘t Hoen, J.H. Frederik Falkenburg, Arnaud Zaldumbide, Marieke Griffioen

**Affiliations:** 1 Department of Hematology, Leiden University Medical Center, Leiden, the Netherlands; 2 Department of Cell and Chemical Biology, Leiden University Medical Center, Leiden, the Netherlands; 3 Department of Human Genetics, Leiden University Medical Center, Leiden, the Netherlands; 4 Department of Medical BioSciences, Radboud University Medical Center, Nijmegen, the Netherlands

**Keywords:** ribosome profiling, cryptic antigen, cancer immunology, T cell antigen, minor histocompatibility antigen

## Abstract

T cell-based immunotherapies targeting antigens on tumor cells have shown efficacy as anti-cancer treatments. While neoantigens are created by somatic mutations acquired during tumorigenesis, allogeneic stem cell transplantation as treatment for hematological malignancies exploits minor histocompatibility antigens encoded by genetic differences between patients and donors. Screening methods to predict neoantigens and minor histocompatibility antigens typically consider only conventional antigens created by nonsynonymous mutations or polymorphisms coding for amino acid changes in canonical open reading frames (ORFs). However, unconventional ORFs encoding peptides outside the known human proteome also provide an important source of cryptic antigens targeted in anti-tumor responses. Here, we used the recently expanded repertoire of human leukocyte antigen (HLA) class I-restricted minor histocompatibility antigens identified in patients treated with allogeneic stem cell transplantation by a method unbiased regarding the type of antigen to explore the sensitivity of ribosome profiling to detect ORFs for different types of T cell antigens. Ribosome profiling showed high sensitivity to detect upstream ORFs for cryptic antigens similar to canonical ORFs for conventional antigens, while cryptic antigens in out-of-frame ORFs and ORFs in long non-coding RNAs were largely missed. In conclusion, ribosome profiling shows variable sensitivity to detect ORFs for canonical and different types of cryptic T cell antigens.

## Graphical abstract

**Figure:**
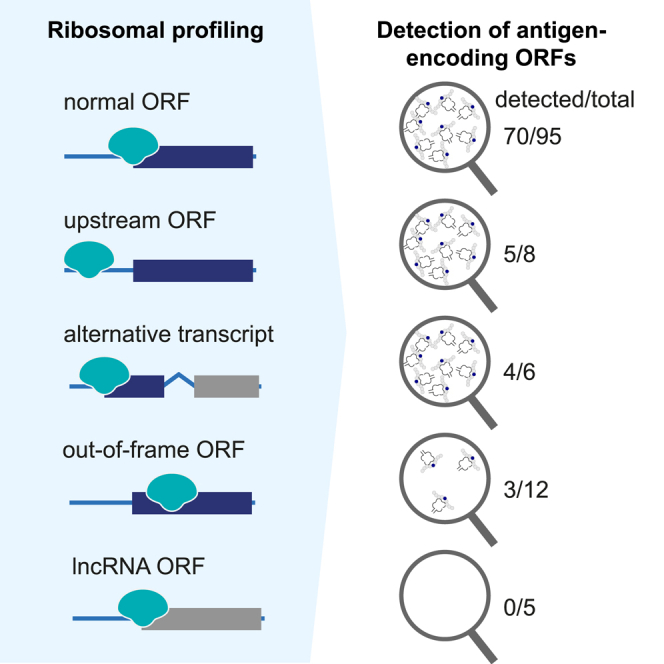

## Teaser

Griffioen and colleagues used HLA I-restricted minor histocompatibility antigens to explore the sensitivity of ribosome profiling to detect open reading frames (ORFs) for canonical and cryptic T cell antigens. Ribosome profiling showed a higher sensitivity for the detection of ORFs encoding cryptic antigens in upstream and alternative transcript ORFs than out-of-frame or lncRNA-ORFs.

## Introduction

Cancer may be cured by immunotherapeutic approaches in which T cells are stimulated to target antigens on tumor cells. Different types of antigens are employed, such as lineage-restricted autoantigens, tumor-associated antigens, and neoantigens.^1^ Neoantigens, which are created by somatic mutations and presented by human leukocyte antigen (HLA) molecules on the surface of cancer cells, are recognized as non-self by T cells. Minor histocompatibility antigens (MiHAs) are targeted by donor-derived T cells in patients treated with allogeneic stem cell transplantation for hematological malignancies. Similar to neoantigens, MiHAs are HLA-binding peptides on patient cells that are foreign to the donor-derived immune system.^2^ While somatic mutations create neoantigens, MiHAs are encoded by genetic differences between patients and stem cell donors.

Prediction methods for neoantigens and MiHAs are based on HLA-binding algorithms or immunopeptidomics and require validation of candidates by antigen-specific T cells. These reverse (antigen-to--T cell) approaches generally search for conventional antigens created by nonsynonymous mutations or polymorphisms coding for amino acid (aa) changes in normal open reading frames (ORFs) of canonical proteins.^3^^,^^4^ However, antigens can also be encoded by (non)synonymous variants in ORFs alternative to canonical protein-coding ORFs (internal out-of-frame ORFs) or variants in 5′ or 3′ untranslated transcript regions, alternative ORFs, or long non-coding RNAs (lncRNAs). To detect ORFs for these cryptic antigens, start sites in actively translated transcripts can be identified by ribosome profiling (Ribo-seq). Others performed Ribo-seq^5^^,^^6^^,^^7^^,^^8^ or used whole-transcriptome RNA sequencing data translated in all reading frames,^9^ showing that peptides from non-canonical ORFs are abundantly present in the immunopeptidome. However, the sensitivity of Ribo-seq, defined by the proportion of ORFs detected by Ribo-seq out of all ORFs for different types of true T cell antigens, has not yet been explored.

In contrast to reverse strategies, forward (T cell-to-antigen) approaches search for antigen-specific T cells and subsequently identify their targets. Antigens identified by forward approaches are not biased by selection steps for specific types of antigens, predicted HLA-binding, gene expression, or other features. By exploring the sensitivity of ribosome profiling to detect ORFs for known T cell antigens identified by forward strategies, relevant insight into the potential use of this technique to identify T cell antigens by reverse strategies can be acquired.

**Figure 1:**
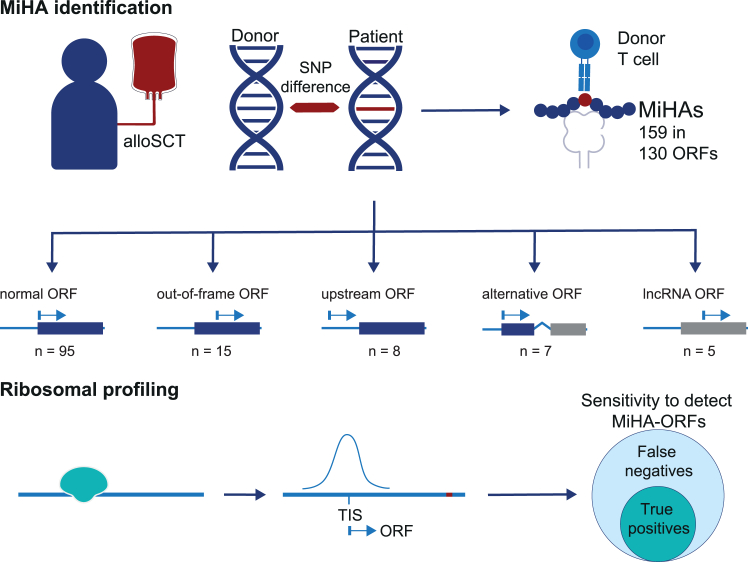
Outline of the study to explore the sensitivity of ribosome profiling to detect ORFs for conventional and cryptic MiHAs HLA class I-restricted MiHAs are T cell antigens targeted in patients treated with allogeneic stem cell transplantation (alloSCT; top). One-quarter of all MiHAs are encoded by unconventional ORFs and were identified by genome-wide association study (GWAS), a method unbiased regarding the type of antigen. The repertoire of 159 MiHAs encoded in 130 ORFs, which contains antigens translated in canonical protein-coding ORFs (*n* = 95) as well as cryptic antigens translated in out-of-frame (*n* = 15), upstream (*n* = 8), alternative (*n* = 7), and lncRNA (*n* = 5) ORFs, has been used to explore the sensitivity of Ribo-seq, defined by the proportion of ORFs detected by Ribo-seq out of all ORFs for different types of true T cell antigens (bottom).

Here, we used HLA class I-restricted MiHAs identified in patients treated with allogeneic stem cell transplantation by forward strategies.^10^ This MiHA repertoire has recently been expanded to 159 antigens, including both conventional and cryptic antigens. Most MiHAs have been discovered by genome-wide association screenings by measuring the reactivity of isolated T cell clones against SNP-genotyped Epstein-Barr virus-transformed B lymphoblastoid cell lines (EBV-LCLs). Reactivity patterns of T cell clones were tested for association with polymorphisms, and peptide candidates were subsequently validated as MiHAs. The 159 MiHAs are encoded by 130 ORFs, 35 (27.7%) of which are cryptic, corresponding to 15 out-of-frame ORFs, 8 upstream ORFs (uORFs), 7 alternative ORFs, and 5 lncRNA-ORFs.^10^ We reasoned that these MiHAs identified by forward strategies provide a valuable source to explore the sensitivity of ribosome profiling to detect ORFs for cryptic T cell antigens (Figure 1).

## Results

To detect MiHA-encoding ORFs (MiHA-ORFs), Ribo-seq was performed using the method and algorithm developed by Thomaidou et al.,^11^ which allows precise mapping of translation initiation start sites (TISs). Initiating ribosomes were arrested with harringtonine, followed by treatment with cycloheximide^11^ on six EBV-LCLs that were included in the 1000 Genomes Project and used for identification of the majority of MiHAs (Table S1; Figure 1).^10^^,^^12^ The six EBV-LCLs were derived from 2 males and 4 females and selected to include at least one MiHA-positive EBV-LCL for all cryptic MiHA-encoding SNPs to ensure that all MiHA-ORFs are represented (Table S1). Of all known 159 MiHAs encoded by 130 ORFs, most MiHAs (143 MiHAs in 118 ORFs) are encoded by ORFs that are present in both patient and donor cells. Detection of TISs for these MiHA-ORFs is independent of the MiHA-encoding SNPs in EBV-LCLs (Table S1). Only a few MiHAs (16 MiHAs in 12 ORFs) are translated in ORFs that are present in patient cells but absent in donor cells. TISs for these MiHA-ORFs can only be detected in EBV-LCLs that are positive for the MiHA-encoding SNPs. These MiHAs include H-Y antigens, which are encoded by polymorphic genes on the Y chromosome relevant in female-to-male transplantations.

In total, 109,821 ORFs with different TISs assigned to 9,121 genes were detected, ranging between 14,721 and 60,262 TISs per EBV-LCL (Table 1). Of all ORFs, the majority of them were initiated by internal in-frame TISs (40.7%), followed by upstream (35.3%) and internal out-of-frame (15.1%) TISs, TISs for canonical proteins (5.5%), and downstream TISs (3.3%). TISs for canonical proteins and internal in-frame TISs led to the longest ORFs of 424 and 263 aa on average, whereas other ORFs were less than 112 aa.

**Table 1:** Detection of ORFs in 6 EBV-LCLs by Ribo-seq

Type of ORF	Detected number of TISs/genes and ORF lengths	EBV-LCLs						Total unique
		HG00114	HG00282	NA12005	NA12044	NA12717	NA12751	
**Detected ORFs**								

Annotated ORFs	number of TISs	2,650	2,160	1,758	4,176	2,335	4,203	6,087
	number of genes	2,612	2,128	1,735	4,079	2,309	4,095	5,079
	average ORF length	394	375	351	415.6	375.3	409	424.5
Out-of-frame ORFs	number of TISs	3,730	2,658	1,900	7,741	2,523	7,876	16,613
	number of genes	1,340	962	763	2,170	947	2,159	3,289
	average ORF length	48.8	46	47.4	41.9	44	43	43.1
Upstream ORFs	number of TISs	11,545	7,902	5,762	20,950	8,329	21,017	38,777
	number of genes	3,688	2,899	2,258	5,319	2,927	5,278	6,471
	average ORF length	108.4	103.1	103.1	109.7	105.1	112	111.8
Internal in-frame ORFs	number of TISs	10,279	6,831	4,895	25,116	7,295	25,227	44,707
	number of genes	3,517	2,572	1,955	5,148	2,769	5,244	6,315
	average ORF length	285.6	273.9	262.9	259.4	269.4	268.9	262.6
Downstream ORFs	number of TISs	729	563	406	2,148	533	1,939	3,637
	number of genes	185	153	111	394	137	383	522
	average ORF length	94	84.9	99.2	82	96.8	79.7	76.8
Total	number of TISs	28,933	20,114	14,721	60,131	21,015	60,262	109,821
	number of genes	5,991	4,854	3,871	7,975	4,943	7,935	9,121
	average ORF length	189.5	182.2	178.5	183.7	184.6	188.3	178.9

**Detected MiHA-ORFs**								

Annotated ORFs	number of TISs/genes	47	40	35	63	41	58	70
	average ORF length	695.7	648.7	560.8	815.4	629.5	784.6	805.9
Out-of-frame ORFs	number of TISs/genes	1	2	1	3	1	3	3
	average ORF length	62	82.5	62	98.7	82	91.7	90.3
Upstream ORFs	number of TISs/genes	3	2	1	4	2	4	5
	average ORF length	64.7	71	30	57.5	42	55	49.8
Alternative ORFs	number of TISs/genes	2	2	1	2	2	4	4
	average ORF length	103.5	93.5	126	90.5	82	173.8	172
lncRNA-ORFs	number of TISs/genes	0	0	0	0	0	0	0
	average ORF length	N/A	N/A	N/A	N/A	N/A	N/A	N/A
Total	number of TISs/genes	53	46	38	72	46	69	82
	average ORF length	625.6	574.8	522.2	723.3	568.2	676.7	701.4

For TISs assigned to more than one transcript, in-frame ORFs or ORFs with the longest peptide sequence were selected. N/A, not applicable.

**Figure 2:**
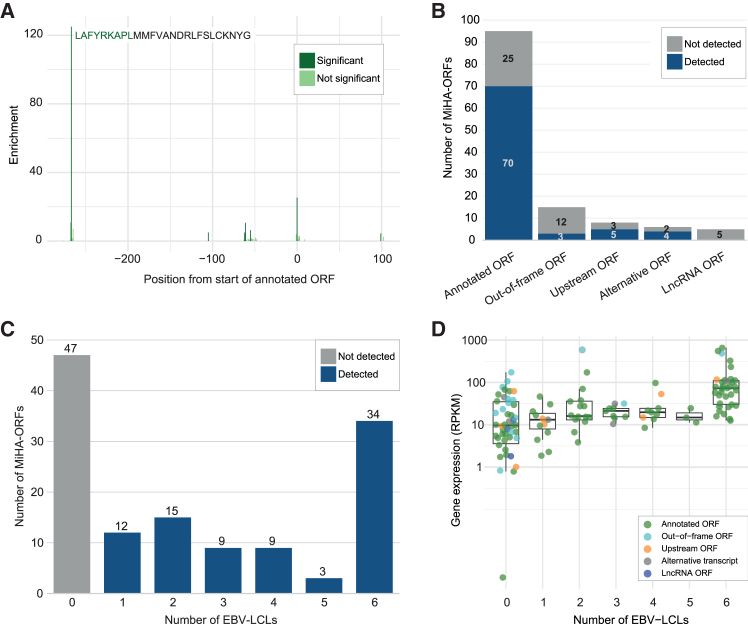
Ribosome profiling to detect ORFs for conventional and cryptic MiHAs Ribo-seq was performed on six EBV-LCLs. (A) Example of a cryptic antigen LB-C12ORF57-1A (LAFYRKAPL) in HG00114, a 9-mer peptide derived from an uORF of 26 aa that starts 267 bp upstream of the canonical protein-coding TIS. Statistically significant and non-significant TISs are shown in dark and light green, respectively. (B) TISs were detected for 82 (63.6%) (blue) of 129 MiHA-encoding ORFs. They primarily coded for conventional MiHAs in annotated ORFs but also cryptic antigens in uORFs, alternative ORFs, and, to a lesser extent, out-of-frame ORFs, but not lncRNA-ORFs. Undetected MiHA-ORFs are shown in gray. As reads were aligned to the human transcriptome, one MiHA encoded by an intron SNP was excluded. (C) Indicated is the number of EBV-LCLs in which MiHA-ORFs were detected. (D) TISs of annotated ORFs for conventional antigens were identified in more EBV-LCLs if average gene expression was high. Two non-detected MiHA-ORFs encoded by lncRNAs are not displayed due to a lack of gene expression data.

As reads were mapped against the transcriptome, one alternative ORF from an intron region could not be detected and was therefore excluded from analysis. In total, 82 (63.6%) of 129 MiHA-ORFs were detected in at least one EBV-LCL (Table 1), as exemplified for a TIS of a cryptic MiHA-ORF (Figure 2A). This TIS created a peptide of 26 aa in an ORF upstream of the canonical protein-coding TIS of *C12orf57*. The first 9 aa represent the MiHA LB-C12ORF57-1A.^10^ Most ORFs were found for conventional antigens, i.e., 70 (73.7%) of 95 MiHAs, and for antigens translated from uORFs (5 of 8 MiHAs) or alternative ORFs (4 of 6 MiHAs) (Figure 2B). In contrast, no TISs were detected for MiHAs encoded by lncRNA-ORFs (*n* = 5), and out-of-frame ORFs were detectable for only 3 (20.0%) of 15 MiHAs, including LB-RPS14-1K translated in an out-of-frame ORF starting upstream of the canonical protein-coding ORF. Detection of MiHA-ORFs varied between 29.5% and 56.6% for each of the six individual EBV-LCLs. Only 34 (29.1%) of 117 MiHA-ORFs present in all six EBV-LCLs were detected in all EBV-LCLs (Figure 2C).

We next investigated whether the sensitivity to detect MiHA-ORFs by Ribo-seq may correlate with gene expression. We compared the average expression of MiHA-encoding genes in the EBV-LCLs as reported by GEUVADIS^13^ to the number of EBV-LCLs in which MiHA-ORFs were found (Figure 2D). All 34 conventional MiHA-ORFs detected in all six EBV-LCLs were found in genes with expression above 10 RPKM compared to only 7 (28.0%) of 25 conventional antigens not detected in any EBV-LCL, indicating a correlation between gene expression and detection of conventional MiHA-ORFs by Ribo-seq. However, despite gene expression above 10 RPKM for 12 MiHAs encoded in out-of-frame ORFs, only 3 (25%) antigens were found by Ribo-seq.

## Discussion

Here, we showed that Ribo-seq detected ORFs for cryptic antigens in uORFs or alternative ORFs with high sensitivity similar to canonical protein-coding ORFs for conventional antigens. The sensitivity to detect annotated ORFs, uORFs and alternative ORFs in at least one of six EBV-LCLs was 72.5%. However, the sensitivity to detect these ORFs reliably in all EBV-LCLs decreased to 30.3%, with the number of detected MiHA-ORFs correlating with sequencing depth for individual EBV-LCLs. Therefore, for cryptic antigens in uORFs and alternative ORFs, Ribo-seq appears to be a reasonable method to filter for peptides in detected ORFs without discarding many true positives. For annotated protein-coding ORFs, we demonstrated that the sensitivity of Ribo-seq was dependent on gene expression. To detect ORFs in low-expressed genes, deeper sequencing may increase the sensitivity of Ribo-seq. However, for antigens in out-of-frame ORFs, the largest group of cryptic MiHAs, our Ribo-seq method is insensitive even if encoding transcripts are abundantly expressed. Insensitive detection of out-of-frame ORFs may be caused by convoluted signals from two reading frames,^14^ for which other Ribo-seq protocols using different inhibitors or algorithms may be more sensitive.^15^ Although the number of MiHAs in lncRNA-ORFs is low, no TISs were detected for these ORFs, suggesting a low sensitivity of Ribo-seq to detect lncRNA-ORFs.

Despite the risk of discarding true positives, Ribo-seq may be considered to increase the specificity of antigen identification by reducing the number of false positives. Bioinformatic approaches to predict cryptic T cell epitopes often result in numerous false positives. These numbers can be reduced by immunopeptidomics, but the technique requires high cell numbers and may fail to detect epitopes with low cell surface expression that are clinically relevant.^16^ Moreover, the number of false positives remain relatively high if mass spectra need to be matched against long lists of cryptic sequences.^17^^,^^18^ As an additional or alternative approach, ribosome profiling can be performed. Similar to gene expression profiling, ribosome profiling requires lower cell numbers and is independent of HLA typing. Although same-sample analysis is preferable, ribosome profiling data from other samples can also be used. This can be particularly useful to reduce the number of cryptic candidates for neoantigens, MiHAs, (lineage-restricted) autoantigens,^11^ or tumor-associated antigens^19^ for patients with different HLA types if no or limited material is available.

In conclusion, using a large collection of HLA class I-restricted MiHAs identified by forward approaches that are unbiased by prediction algorithms, we explored the sensitivity of Ribo-seq to detect ORFs for canonical and cryptic antigens. The data demonstrated that cryptic antigens in uORFs and alternative ORFs were found with higher sensitivity than antigens in out-of-frame or lncRNA-ORFs, indicating that Ribo-seq may be particularly useful to screen for these antigen types as potential targets for immunotherapy.

## Materials and methods

Ribo-seq was performed on six EBV-LCLs from the 1000 Genomes Project (HG00114, HG00282, NA12005, NA12044, NA12717, NA12751) as described previously.^11^ Each EBV-LCL (20 million cells) was treated with 2 μg/mL harringtonine for 30 min, followed by the addition of 100 μg/mL cycloheximide, and resuspended in lysis buffer supplemented with 100 μg/mL cycloheximide. Ribosome complexes were isolated by density purification. Small RNA molecules were isolated using the NucleoSpin miRNA kit (Bioké, Leiden, the Netherlands), followed by RNA-PAGE. Universal linkers were added after dephosphorylation. After reverse transcription, cDNA was circularized and, after ribosomal RNA depletion, barcoded and sequenced. Reads were aligned to the human transcriptome (ENSEMBL_GRCh38.99). P-site offsets were computed using an enrichment method and significance testing with a negative binomial regression model as described.^11^ To analyze gene expression, RNA sequencing data of selected EBV-LCLs were retrieved from GEUVADIS.^13^

## Data and code availability

Raw data are available in the European Genome-phenome Archive under EGA: EGAS50000000322.

## Acknowledgments

This work was supported by the 10.13039/501100004622Dutch Cancer Society (10713). Distribution of EBV-LCLs was done under the 10.13039/100011102European Commission 7th Framework Program (FP7) (261123; GEUVADIS). The authors thank Roderick C. Slieter and Davy Cats for support with data analysis and management.

## Author contributions

K.J.F., S.T., J.H.F.F., A.Z., and M.G. designed research. K.J.F., S.T., A.R.v.d.S., and M.v.d.M. performed research. P.A.C.t.H. contributed essential materials. K.J.F., S.T., J.H.F.F., A.Z., and M.G. analyzed data. K.J.F., S.T., P.A.C.t.H., J.H.F.F., A.Z., and M.G. wrote the manuscript. All authors reviewed the manuscript.

## Declaration of interests

The authors declare no competing interests.

## Supplemental information

Table S1. Overview of HLA I-restricted MiHAs investigated for detection of MiHA-ORFs by Ribo-seq The six EBV-LCLs selected for ribosome profiling have been tested for recognition by MiHA-specific Tcell clones in IFNγ ELISA (column N; Fuchs et a. *Blood* 2024). Asterisks indicate results where Tcell recognition was absent, whereas the EBV-LCL was positive for the MiHA, probably due to weak reactivity of the Tcell clone, or results where Tcell recognition was observed, although the EBV-LCL was negative for the MiHA, potentially due to crossreactivity of the Tcell clone. n.d. = not determined; n.a. = not applicable.
